# Cerebral Tuberculosis After Therapy With Adalimumab for Hidradenitis Suppurativa: A Rare Case

**DOI:** 10.7759/cureus.52267

**Published:** 2024-01-14

**Authors:** Francisca Martins, Alexandra Rodrigues, João Fonseca Oliveira, Rui Malheiro, Luís Cerqueira

**Affiliations:** 1 Internal Medicine, Centro Hospitalar Universitário de Lisboa Central, Lisbon, PRT; 2 Neuroradiology, Centro Hospitalar Universitário de Lisboa Central, Lisbon, PRT

**Keywords:** hidradenitis suppurativa, tuberculosis, immunosuppression therapy, iatrogeny, central nervous system tuberculosis, hidradenitis suppurativa treatment, adalimumab (humira)

## Abstract

Hidradenitis suppurativa (HS) is a chronic inflammatory skin disease with limited therapeutic options. Adalimumab, an anti-tumor necrosis factor-alpha (TNF-α) monoclonal antibody, was the first biological agent approved for the treatment of moderate to severe HS. Tuberculosis (TB) is a highly prevalent global public health problem, affecting individuals worldwide. Continuous immunosuppression from TNF-α treatment increases the risk of TB development. Isolated neurotuberculosis, in the absence of other symptoms, emerges as a rarely observed infection pattern in such patients.

We present a case of a 23-year-old woman with severe HS undergoing treatment with adalimumab. After two years, she developed a pronounced occipital tension headache, constant nausea, and persistent fever. The patient's latent TB status was unknown without annual screening. Subsequent magnetic resonance imaging revealed a lesion in the cerebellar vermis. Immunosuppressive therapy was suspended and an etiological study was conducted; the only positive result was the interferon-gamma release assay. Empirically, antituberculosis treatment and prednisolone were initiated, leading to clinical and neurological improvement. After one year of treatment, symptoms resolved without neurological sequelae.

This case highlights the importance of vigilant monitoring before, during, and after immunosuppressive treatment. Early recognition, discontinuation of anti-tumor necrosis factor medications, and appropriate management of TB are crucial to prevent complications.

## Introduction

Hidradenitis suppurativa (HS) is a chronic inflammatory skin disorder characterized by recurrent, painful, and deep-seated abscesses, primarily in areas with apocrine sweat glands, such as the axillae, groin, buttocks, and infra-mammary regions [1-3]. This condition typically presents with painful nodules, sinus tracts, and scarring, often leading to considerable morbidity and reduced quality of life [1,2]. The global prevalence of HS is estimated to range from 0.00033% to 4.1% [3]. Women are disproportionately affected, with a two to three times higher incidence compared to men [1]. The highest occurrence of HS is observed in patients aged between 18 and 44 years [4]. Management options include a combination of medical therapies, such as antibiotics, immunosuppressants, and biologics [5,6], associated with lifestyle modifications, as well as surgical interventions, including incision and drainage, wide local excision, or laser therapy [5]. The prognosis of HS can vary widely among individuals, with some experiencing chronic, relapsing disease, while others achieve prolonged periods of remission [1,2]. Early diagnosis and comprehensive management are crucial for optimizing outcomes in affected patients.

Anti-tumor necrosis factor-alpha (TNF-α) is a pro-inflammatory cytokine with a significant role in the pathogenesis of chronic inflammatory diseases and other immune-mediated conditions [5]. TNF-α increases endothelial permeability, enhances leukocyte migration, and activates neutrophils and eosinophils functionally. Consequently, it is involved in a range of adaptive immune responses, including granuloma formation, phagosome development, macrophage activation and differentiation, and the immune response against viral pathogens [7].

Adalimumab, a TNF-α inhibitor, has shown promise in the treatment of HS, a debilitating chronic skin condition [5]. By targeting the pro-inflammatory cytokine TNF-α, adalimumab can help mitigate the inflammatory processes associated with HS, reducing the frequency and severity of painful abscesses, sinus tracts, and scarring [5,6]. It is used for moderate to severe HS cases that are refractory to conventional therapies [5], demonstrating the potential to improve quality of life and induce long-term disease remission in some individuals [5-9]. Adalimumab is an important therapeutic tool in the management of this challenging condition; however, like other biologics, its use comes with considerations for potential side effects and increases susceptibility to severe and opportunistic infections, including tuberculosis (TB) [6,7].

TB remains a leading global cause of mortality [10]. Despite a decline in new cases in Portugal, the country still maintains the highest TB incidence rate in Western Europe. In 2021, Portugal reported a total of 1,513 TB cases, equating to a notification rate of 14.6 cases per 100,000 residents. Notably, the Northern region and the Lisbon and Tagus Valley region continue to exhibit the highest incidence rates: 17 cases per 100,000 inhabitants [11]. Severe forms of TB, i.e., disseminated, meningeal, or involving the central nervous system (CNS), account for 5.3% of the total number of cases [11].

This report delineates a case of a female patient afflicted with HS, who, two years after commencing adalimumab therapy, manifested CNS TB. Although CNS TB is a known consequence of TNF-α therapy, it is crucial to maintain awareness of latent tuberculosis infection (LTBI), particularly in regions with high prevalence. This vigilance is essential to reduce adverse outcomes. This report offers a comprehensive analysis encompassing the clinical facets, diagnostic considerations, therapeutic approaches, and prognostic outlook for this particular presentation.

## Case presentation

This case described a 23-year-old woman medical student in Portugal. She had a medical history dating back to the age of 12 years, marked by the presence of HS. In early 2019, she started adalimumab due to extensive suppurative lesions with fistulous tracts in various areas of her body (medial thighs, inguinal region, pubic mound, gluteal region, axillary, and inframammary region). In October 2020, she developed varicella-zoster virus (VZV) affecting the trigeminal nerve and was treated with valacyclovir and corticosteroids. Over the next four months, she experienced neuropathic facial pain, which improved and eventually resolved.

**Figure 1:**
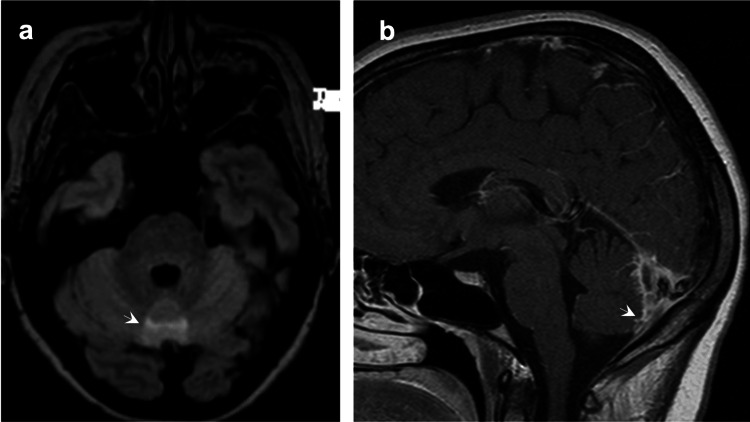
(a) T2WI-FLAIR shows a subtle area of hyperintensity in the posterior region of the cerebellar vermis (arrow). There is no evidence of restricted diffusion, suggesting probable vasogenic edema. (b) Contrast-enhanced T1WI reveals pachymeningeal diffuse thickening and enhancement in the falcotentorial posterior region (arrow). T2WI: T2-weighted imaging; FLAIR: fluid-attenuated inversion recovery; T1WI: T1-weighted imaging.

In September 2021, the patient presented with a recurrence of neuropathic pain in the ophthalmic and maxillary branches of the trigeminal nerve, accompanied by low-grade fever, nausea, malaise, and tensional occipital pain. Her primary care physician initially diagnosed the condition as post-herpetic neuritis. However, the patient felt that her symptoms were atypical and requested a cerebral magnetic resonance imaging (MRI) that revealed a lesion in the superior vermis of the cerebellum, involving the cuneus, hyperintense on T2-weighted images (WI) and hypointense on T1-WI, with minimal mass effect and no contrast enhancement (Figure 1).

Following the referral to the internal medicine department for further evaluation, the patient presented with an intense occipital tension headache (7 out of 10 on the numerical pain scale), constant nausea, and daily persistent fever. There were no other constitutional symptoms or meningeal signs. Laboratory tests revealed mild normocytic, normochromic anemia, leukocytosis with neutrophilia, elevated lactate dehydrogenase, C-reactive protein of 81.4 mg/L, and an erythrocyte sedimentation rate of 89 mm/h. A lumbar puncture showed clear cerebrospinal fluid (CSF) with mononuclear cells, normal glucose, elevated protein, negative viral polymerase chain reaction (PCR), and negative *Mycobacterium tuberculosis* deoxyribonucleic acid (DNA). CSF samples were examined using Ziehl-Neelsen (ZN) stain for acid-fast bacilli, Gram stain for bacteria, India ink preparations for fungi, and an antigen test for *Cryptococcus neoformans*. All results were negative. The patient was unaware of the result of the interferon-gamma release assay (IGRA) test for TB before starting treatment with adalimumab, and she did not undergo an annual check-up. As a child, she had been vaccinated with Bacillus Calmette-Guérin (BCG).

**Figure 2:**
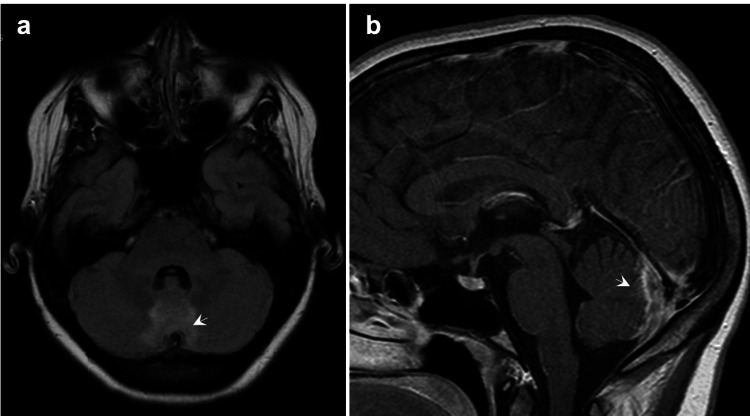
Two weeks later, (a) T2WI shows an increase in hyperintensity in two-thirds of the superior vermis and adjacent areas, indicating an increase in vasogenic edema (arrow). (b) Contrast-enhanced T1WI shows an increase in irregular pachymeningeal enhancement adjacent to the cerebellar vermis, without restricted diffusion (arrow). T2WI: T2-weighted imaging; T1WI: T1-weighted imaging.

In the following days, as cultural results were pending, the patient’s headaches and nausea worsened despite receiving antiviral empiric therapy with intravenous acyclovir. A cerebral MRI, conducted two weeks later, indicated worsening infiltrative changes in the dura mater, primarily in the torcular region, vermis, and right occipito-polar region. It suggested an inflammatory process, possibly granulomatous, with the involvement of the cerebral venous sinuses (Figure 2). The IGRA test returned positive.

**Figure 3:**
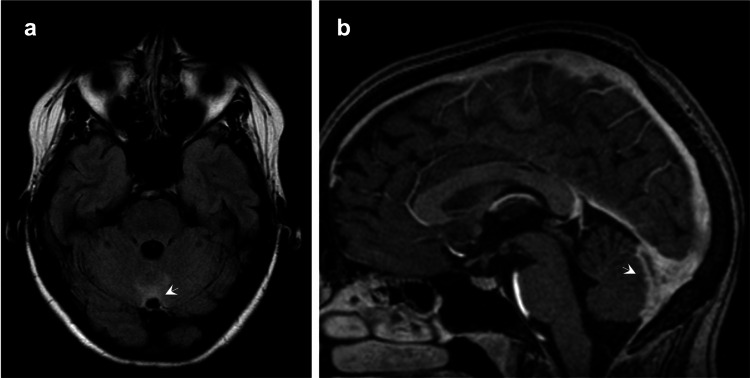
Five weeks after the initial cerebral MRI, (a) T2WI-FLAIR shows a reduction in edema in the vermis (arrow). (b) Contrast-enhanced T1WI reveals a decrease in pachymeningeal enhancement (arrow). T2WI: T2-weighted imaging; FLAIR: fluid-attenuated inversion recovery; T1WI: T1-weighted imaging.

Given the deteriorating condition, empirical anti-tuberculosis treatment was initiated with isoniazid, rifaximin, ethambutol, pyrazinamide, and pyridoxine, alongside prednisolone. Within a week, her symptoms improved significantly. Five weeks after starting treatment, the lesion had regressed in MRI (Figure 3).

**Figure 4:**
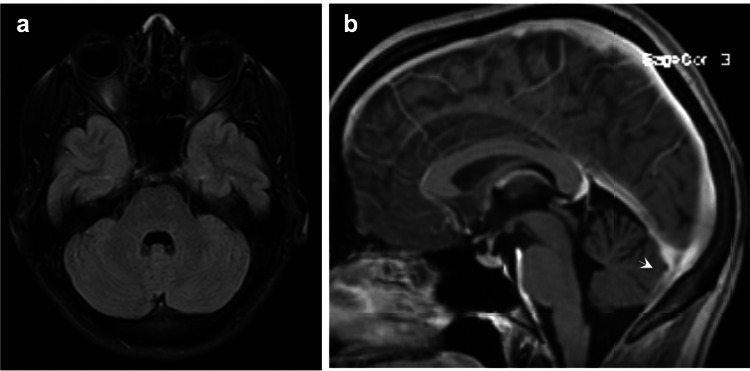
One year after the initial cerebral MRI, (a) T2WI shows complete resolution of the signal alteration in the vermis (arrow), medial cerebellar hemispheres, and occipito-polar region (edema). (b) Contrast-enhanced T1WI reveals reduced thickening and pachymeningeal enhancement previously observed in the region of the torcula (arrow). T2WI: T2-weighted imaging; T1WI: T1-weighted imaging.

After 42 days, mycobacterial cultures turned out negative. She completed 12 months of anti-tuberculosis treatment, and a follow-up MRI one year after the initiation of treatment revealed a significant improvement in the dural thickening and the disappearance of the previously observed imaging abnormalities (Figure 4). The patient showed a favorable response to anti-tuberculosis therapy, with significant clinical and radiological improvement, without neurological sequelae.

## Discussion

Adalimumab is a fully human IgG1 monoclonal antibody that specifically targets TNF-α. It is currently approved for the treatment of various inflammatory diseases, including moderate to severe HS [12,13]. While its ability to neutralize TNF-α contributes to disease control through immunomodulation and immunosuppression, it can also render individuals more susceptible to infections [7,12-14].

In the context of LTBI, mycobacteria can persist within macrophages [9]. Anti-TNF-α agents have the potential to trigger the reactivation or dissemination of *Mycobacterium tuberculosis*, leading to active TB [7-9]. Conversely, TNF-α has not been implicated in the innate immune response against extracellular bacterial pathogens [9,12]. Furthermore, this therapy theoretically increases susceptibility to other pathogens such as bacteria (*Listeria monocytogenes* or *Salmonella* spp.) and viruses (hepatitis B virus, VZV, and human polyomavirus John Cunningham) [7,12].

In the case of this patient, she initially presented with a VZV infection. Despite being on immunosuppressive therapy, vaccination is not recommended due to her age [12]. This is because even under these conditions, the infection is rare within her age group. A higher frequency is observed in patients over 60 years on anti-TNF-α therapy, although the risk in this age range, compared to the general population, is not universally agreed to be increased [7,12,14].

Among patients with TB, approximately 1% to 5% are complicated by CNS TB. In regions with low TB prevalence, such as North America and Western Europe, extrapulmonary manifestations of TB are primarily observed in adults with reactivated disease, with CNS TB, particularly in the form of meningitis, being the predominant presentation [15]. Following the inhalation of aerosol droplets containing *M. tuberculosis*, the host may either immediately eliminate the organism, develop primary active disease, remain with LTBI, or progress to active disease after a period of latent infection [12,14]. Among individuals with LTBI and no underlying medical conditions, reactivation disease occurs in approximately 5% to 10% of cases [15]. The risk of reactivation significantly increases in immunosuppressed patients, particularly those on anti-TNF-alpha therapy, estimated to be two to four times higher. While TB can result from new exposure, in the context of immunosuppressive therapy, it typically stems from the reactivation of latent infection [16].

To mitigate this risk, screening is recommended for patients who are candidates to initiate biological immunosuppression, both before its commencement and subsequently on an annual basis or sooner if there is exposure to a patient with active TB [16]. The screening process involves, first and foremost, ruling out active disease through symptom inquiry, assessment of signs consistent with TB disease, and chest radiography. Subsequently, it involves the exclusion of LTBI by inquiring about past TB and/or potential high-risk contacts [12,14,16]. There is no gold standard analytical method for diagnosing LTBI [14,16]. These tests, in addition to relying on an intact immune system, which may compromise their interpretation in patients under immunosuppressive therapy, are imperfect in assessing the risk of progression to TB disease. Among the available tests, IGRA exhibits higher sensitivity compared to the tuberculin skin test (TST) [12,14,16]. In the absence of past TB treatment, a previously positive IGRA before the initiation of therapy is an indication for LTBI treatment, typically involving monotherapy for four to nine months [14]. In the case of our patient, we did not have access to the results of pre-treatment tests and we know that the annual control was not carried out.

Diagnosing active TB in these patients can be challenging since symptoms related to cellular immune response (e.g., fever) and corresponding signs of inflammation (pulmonary infiltrates) may be absent or reduced [17]. Diagnostic investigation in these patients should follow the traditional approach, involving the collection of microbiological specimens for direct examination (which has lower sensitivity in immunosuppressed patients), culture, and nucleic acid amplification tests for TB and other potential opportunistic diseases [14]. In patients on anti-tumor necrosis factor (TNF) medications, the disease presents as extrapulmonary or disseminated in 25-48% of cases [17], with central nervous system involvement still being rare, primarily manifesting as meningitis [17-19].

Neurotuberculosis can manifest in various ways, categorized as extra-axial and intra-axial presentations. Extra-axial manifestations include the most common tuberculous meningitis (leptomeningitis) and the rare tuberculous pachymeningitis. Intra-axial manifestations encompass intracranial tuberculous granuloma (tuberculoma), focal tuberculous cerebritis, intracranial tuberculous abscess, tuberculous rhombencephalitis, and tuberculous encephalopathy [18]. The cavernous sinus, middle cranial fossa floor, tentorium, and cerebral convexity are commonly involved sites [19]. Differential diagnoses for neurotuberculosis include other infectious causes of meningitis/encephalitis, neurosarcoidosis, meningioma, as well as neoplastic conditions such as metastasis and lymphoma. Differential diagnosis is made through lesion biopsy [18].

Considering the patient's clinical features, which include immunosuppression due to anti-TNF therapy, the absence of a personal history of TB, a positive IGRA result (despite prior BCG vaccination, which may influence the outcome), and the occupational exposure risk linked to her profession, a decision was made to commence empirical treatment and monitor the response to therapy. Chemoprophylaxis has been a topic of discussion for at-risk groups; however, there is no consensus recommending its implementation in patients with negative IGRA [20].

Timely identification with an accurate diagnosis, discontinuation of anti-TNF medications, and prompt initiation of treatment are crucial measures for achieving successful therapy [12,14]. These steps can help prevent disease exacerbation, minimize the risk of neurological complications, and reduce the potential for long-term health effects and mortality [12,14,16].

## Conclusions

This case of neurotuberculosis in a young individual following immunosuppressive treatment for HS underscores the critical importance of early screening for opportunistic diseases in patients undergoing immunosuppressive therapies. Notably, the initial unknown serological status before treatment initiation and the preceding episode of trigeminal herpes zoster, which initially obscured the patient's complaints, add complexity to the diagnostic challenge. The manifestation of cerebral TB in this context highlights the potential reactivation of latent infections that can occur when the immune system is compromised, emphasizing the need for vigilant monitoring before, during, and after immunosuppressive treatment.

Furthermore, this case reinforces the significance of thoroughly assessing and investigating patients’ complaints, particularly those related to new neurological symptoms, irrespective of the initial pathology. The successful management of this case underlines the importance of adopting a holistic approach in medical practice. Careful assessment of underlying conditions, diligent monitoring during immunosuppressive treatment, and comprehensive investigation of patient complaints are pivotal in preventing and managing complications stemming from opportunistic diseases.
